# Users’ perceptions about receiving personalized depression risk information: findings from a qualitative study

**DOI:** 10.1186/s12888-021-03590-8

**Published:** 2021-11-18

**Authors:** Heidi Eccles, Doaa Nadouri, Molly Nannarone, Bonnie Lashewicz, Norbert Schmitz, Scott B. Patten, Douglas G. Manuel, JianLi Wang

**Affiliations:** 1 The Royal’s Institute of Mental Health Research, University of Ottawa, Ottawa, Canada; 2 Department of Community Health Sciences, Cumming School of Medicine, University of Calgary, Calgary, Canada; 3 Department of Psychiatry, Faculty of Medicine, McGill University, Montreal, Canada; 4 School of Epidemiology and Public Health, Faculty of Medicine, University of Ottawa, Ottawa, Canada; 5 Department of Community Health and Epidemiology, Faculty of Medicine, Dalhousie University, 5790 University Ave. Halifax, Nova Scotia, B3H 1V7 Canada

## Abstract

**Objectives:**

To understand users’ perceptions about receiving their personalized depression risk score and to gain an understanding about how to improve the efficiency of risk communication from the user perspective.

**Methods:**

A qualitative study embedded in a randomized controlled trial (RCT) on evaluating the impact of providing personalized depression risk information on psychological harms and benefits. The participants (20 males and 20 females) were randomly selected from the intervention arm of the RCT after the 12-month assessment. The qualitative interviews were conducted through telephone, audio recorded and transcribed verbatim. We conducted a content analysis to describe the content and contextual meaning of data collected from participants.

**Results:**

The first theme explained the motivation for receiving a risk score. Most participants chose to receive their personalised depression risk score with the goal of improving their self-awareness. The results revealed three sub-themes surrounding perceptions and implication of receiving their risk score: positive, negative, and neutral. Most participants found that receiving their score was positive because it improved their awareness of their mental health, but some participants could see that some people would have negative feelings when getting the score causing them to be more likely to get depression. The final theme focussed on improvements including: the best delivery methods, having resources and strategies, and targeting younger people.

**Conclusion:**

The most significant motivation for, and benefit of receiving one’s personalized depression risk score was improved awareness of one’s mental health. A comprehensive risk communication program may improve the uptake and maximize the impact on behavior changes and risk reduction.

## Introduction

Depression is a prevalent affective disorder, and it accounts for 4.3% of all global disability life years [1]. In Canada, people aged 15 and older have a 4.7% annual prevalence of depression, which lead to an economic burden of over $12 billion per year across Canada [2]. Depression has a drastic effect on an individual’s physical and mental health leading to psychophysical diseases, disability and increased mortality risk. Successful implementation of self-help strategies relies on researchers’ capability of estimating one’s baseline risk and effectively communicate such risk information to the users. Multivariable risk predictive models have been used to estimate risk which is the probability of developing a health condition over a specific time period. The estimated risk has a number of applications [3]. One application is risk communication; the risk information is communicated to users to inform decision making, raise awareness about health status, and prompt positive changes to health-related behaviours (e.g., self-management, initiation of treatment) to reduce their risk. There have been a number of studies on risk predictive models and risk communication in various medical disciplines [4] including cardiology, [5] oncology [6, 7] and genetics [8, 9]. Compared to these medical disciplines, risk predictive analytic research for mental health problems is scarce, with few risk predictive models for major depressive episode (MDE) in the primary care settings [10, 11], general population [12, 13], for psychosis in clinical samples [14, 15], and for suicide [16, 17].

In the field of mental health, self-help strategies have been commonly used to manage symptoms of depression [18]. The promotion of effective self-help strategies to the general public as an early intervention strategy has been recommended as a method to reduce the burden of depression without placing a burden on healthcare resources [19]. However, changing behaviours to improve mental health and encouraging help-seeking have been very challenging [20]. Based on the experience of other medical disciplines, personalized depression risk communication may offer a unique strategy for promoting behaviour change.

Using longitudinal data from a representative sample of the Canadian general population, we previously developed sex-specific multivariable risk predictive algorithms for MDE [12]. The risk predictive models include demographic and socioeconomic characteristics, personal and family history of MDE, current health status, ongoing stress and experience of childhood traumatic events. It also estimates the risk of having an MDE in the next 4 years [12]. To understand the potential psychological harms and benefits associated with providing personalized depression risk information, we conducted a mixed methods randomized controlled trial (RCT) in individuals who were at high risk of having an MDE [21, 22]. It is important to understand the perception people have of receiving a score to predict their risk of depression therefor our aim is to understand users’ views about personalized depression risk score, the way in which it was delivered, and how its delivery can be improved.

## Methods

### Broader study context

This grounded theory study was embedded in a mixed methods RCT. The RCT was conducted between April 2018 and May 2020 included 712 participants across 10 provinces. The target population were adults who were at high risk of having MDE in the next 4 years. Eligible participants were randomized into the intervention group (receiving personalized depression risk information) and control group (receiving generic mental health information). Participant were interviewed via telephone at baseline, 6- and 12-month. These interviews were to quantitatively assess the effectiveness of the intervention. The intervention group received their depression risk score at the end of each interview. Detailed methodological information about the trial can be found in previous publications [21, 22].

### Qualitative descriptive study sample

Participants for the qualitative interviews were recruited from the intervention group of the RCT after the 12-month follow-up quantitative assessment. Of the original 712 participants, 306 were in the intervention group. Participants were eligible if 1) they had been given their risk score during all follow up interviews (baseline, 6 month and 12 month); and 2) they had finished the 12-month assessment in February 2020. Using that eligibility criteria, 100 of the original 712 were eligible to participate in the qualitative interviews. A total of 54 participants were purposely randomly selected from the eligible participants and contacted for the qualitative interview. All participants gave oral informed consent to participate in the qualitative interviews. This study was approved by the Research Ethics Board at the Royal Ottawa Mental Health Center.

Choosing participants from those who completed the 12-month quantitative assessment in February 2020 (as opposed to the rest of the participants who had completed the 12-month quantitative assessment in August of 2019) was aimed at decreasing the chance of recall bias. The first step of random purposeful sampling was chosen similar to a quantitative recruitment method to be representative of the sample and be similar to the broader quantitative study which included the same number in each gendered group. Then the total sample size was chosen to satisfy the need for code saturation. The sample size of 20 men and 20 women was decided a priori as 20 interviews (per gender group) is a commonly agreed on “more than adequate” number of interviews for achieving code saturation which is occurs when no new codes of content are evident in subsequent interviews [23–25]. Malterud and colleagues [26] affirm that the number of interviews needed to reach saturation to can be found by looking at features of the sample and understanding of the research topic. As this sample is heterogeneous and little is known about the perceptions on personalized risks of depression, the research team deemed 40 interviews total was needed to reach saturation. Therefor the researchers contacted the participants until 40 people (20 men and 20 women) consented to participate in the study.

### Data collection

A semi-structured interview guide was developed to include questions about a) whether they understood its meaning; b) why they chose to receive it; c) their feelings regarding their risk score, and any effects that it may have had on them; d) physical and mental health changes that they may have experienced since their risk score was disclosed; and e) possible improvements to the delivery of depression risk information. Semi-structured interviews were conducted over the telephone by HE and DN from April to May 2020. The interviews were audio recorded and transcribed verbatim.

### Data analysis

Our analysis focused on the impact that receiving personalized depression risk information had on participants; content analysis was performed to describe the content and contextual meaning of the data [27, 28]. Analysis began with two researchers (HE & DN) reading and re-reading the transcribed interviews to gain familiarity with the ideas within the data. They then reduced the data into codes - words or short phrases that describe the content and recurring themes. Codes were then grouped together into categories. HE and DN independently created a coding framework; these coding frameworks were then compared, and any discrepancies were discussed to reach consensus. HE and DN then shared the coding framework with a wider group of mental health researchers who were involved in data collection for the RCT. The revised coding framework was then used by HE and DN to recode the data with guidance from BL, a qualitative research specialist who is part of the broader mixed methods RCT team. BL was also chosen because she was not involved in data collection or initial rounds of analysis.

## Results

Forty of 54 participants (20 males and 20 females) were successfully contacted and agreed to participate. Baseline socio-demographic characteristics of participants are summarized in Table 1. Three main themes emerged from the data. The first theme relates to why participants chose to receive their depression risk scores. The second theme relates to what participants thought of, and did with their risk scores, is also comprised of 3 subthemes that capture: a) positive perceptions and implications; b) negative perceptions and implications; and c) neutral perceptions and implications. This theme overlaps in a mixed-methods paper by the authors to help understand the quantitative results (21). The third theme relates to strategies to improve risk communication, and is comprised of three sub-themes a) varying the format of delivery of personalized risk scores; b) augmenting risk scores with resources, strategies and follow up; and c) targeting young people to increase awareness.

**Table 1:** Baseline characteristics of the participants

Variable	Total (*N* = 40)	Female (*N* = 20)	Male (*N* = 20)
Age mean (range)	36 years (19–64)	37.65 (19–54)	34.2 (22–64)
Marital status			
Married	20	11	9
Single	16	5	11
Divorced or Widowed	4	3	1
Education			
Elementary	1	1	0
High school incomplete	1	0	1
High school complete	3	1	2
College incomplete	2	1	1
College complete	11	6	5
University incomplete	4	2	2
University complete	10	3	7
Graduate degree	8	5	3
Location			
BC	4	3	1
AB	5	3	2
SK	1	1	0
MB	3	2	1
ON	15	5	10
QC	5	3	2
NB	2	1	1
NS	4	2	2
PE	1	0	1
Predicted risk mean (range)	25.0% (6.8–85.4)	27.2 (11.7–85.4)	22.7% (6.8–77.7)
Perceived risk mean (range)	33.2% (0–100)	39.1 (0–100)	25.2 (0–95)
K10 score mean (range)	19.2 (10–36)	20.2 (12–36)	18.2 (10–34)
K10 Score			
20 or above	14	8	6
Below 20	26	12	14

Abbreviations: *AB* Alberta, *BC* British Columbia, *MB* Manitoba, *NB* New Brunswick, *NS* Nova Scotia, *ON* Ontario, *PE* Prince Edward Island, *QC* Quebec, *SK* Saskatchewan

### Theme 1- motivations for receiving a personalized depression risk score

Participants reported being motivated by curiosity and a desire to increase their personal awareness of their mental health. Some participants revealed that they answered the series of risk assessment questions as part of their RCT participation because they were curious to find out their risk score. Others indicated their willingness to gain awareness about their mental health. A male participant commented: “I am prone to a little bit of depression or sadness. So I just wanted a, maybe a professional, maybe not quite assessment but outside view if you will,” (ID 20468, Male) while another male participant said: “taking part in the study and the interviews was a good step in trying to understand [my]self.” (ID 20683, Male).

### Theme 2 – perceptions about the risk score

Participants’ perceptions and implications of having received their risk score are in three sub-themes: positive, negative, or neutral.

#### Positive perceptions and implications

Some participants perceived having received their score as a positive experience. Many said that the personalized risk score was useful in improving self-awareness of mental health and thinking about how mental health can be improved: “It helps you understand where you are in terms of others and maybe that could decide if what you are doing is good or if you need to do something different.” (ID 20552, Male). Some participants noted that obtaining a personalized risk score could provide a reason to think about mental health and to recognize risk. One female participant reflected: “… if you don’t know you are depressed or you don’t understand how depression works and you kind of like go by a normal everyday kind of life thinking that you aren’t depressed and then you feel different. It would be nice for those people to have that insight that they are at risk.” (ID 20475, Female). Although many participants did not consider their risk score as a key factor in changing their behaviours, they did believe that their risk score remained “in the back of [their] head.” One participant said that receiving his risk score prompted some reflection: “it wasn’t always really present for me, but it is something I reflected on occasionally.” (ID 20663, Male).

Other participants spoke of the implications of having received their risk score in terms of changing their behavior. Participants felt validation that their current strategies to improve their mental health were effective,: “You know, I have to start changing some things in my life and then I felt good when I saw that it went down.” (ID 20570, Female). Some participants spoke of having shared their risk score with family and friends, and the benefits of sharing their risk score as part of obtaining support and having open conversations with the people they know. One female participant said, “Because I can discuss it with my family, and they can understand me better.” (ID 20522, Female). Other participants reported believing it was relevant to share their risk score with their healthcare providers. A female participant claimed: “Oh yeah I shared it with my spouse and I talked to my doctor about it as well … My family doctor wasn’t surprised because she knows my family history and you know and she was … just she gave me a run down on things that I could do to help try and improve the outcome, and she is always keeping an eye on my medication and things.” (ID 20592, Female).

#### Negative perceptions and implications

Although nobody conveyed that they personally felt any negative implications, a few participants expressed concern over potential negative impacts that other people might experience in learning their risk scores. Notably, participants were concerned that individuals might be stressed by receiving a high-risk score and that such stress could be exacerbated if an individual had inadequate support systems or coping mechanisms. One female participant described how stress in response to receiving a high score might play out: “sometimes in those situations when you’re already feeling down and you suffer from depression and things can tend to spiral. And I don’t know if that would maybe give him the push they need to get help or if that would just reinforce that action of mindset not good enough to get a low score”(ID 20503, Female), and another participant claimed that “I just think that if there is somebody that’s like more alone in their life and doesn’t have the supports that they need and they give that they are at a high risk of depression or mental health issues it might toy with their emotions …” (ID 20467, Female).

#### Neutral perceptions and implications

Some participants said receiving their personalized depression risk score did not affect them. They indicated an awareness of their risk, yet did not wish to make changes; one male participant commented that “I don’t dwell on it. That’s not making me change anything I am doing, I guess. It is not on the back of my mind.” (ID 20505, Female). Some described already having effective coping strategies in place and not believing they needed to change anything: “Maybe just curiosity and I mean I have received mental health support on an ongoing basis. So knowing those numbers didn’t really specifically encourage me to go and seek help because I go … I receive mental health support on and off.” (ID 20565, Female).

### Theme 3 – strategies for improving risk communication

#### Format of delivering risk scores

The data suggests that having scores delivered through the Iinternet and counselor had many benefits and disadvantages depending on the person. Highlighting the need for multiple delivery methods to suit the varying people. Some participants thought Internet delivery of depression risk scores was the best given the stigma that surrounds mental illness, and that Internet delivery is most accessible. However, other participants did not agree, and pointed out that in cases where the risk score is higher than average, people with unstable mental health might not receive appropriate help if they receive their risk score online. Participants also said that they would question whether they could trust scores they find on the Internet and expressed skepticism including the idea that: “people can say anything online.” (ID 20527, Female). Further, some participants questioned the impact of receiving a score on the Internet noting that a score received on the Internet might not provoke further thought.

Given the sensitive nature of depression, having a person such as a counselor deliver the depression risk score, whether in person or by telephone, was considered valuable. Participants expressed that having a person deliver depression risk scores would be especially important to offset negative consequences of receiving a score as recipients and would be able to ask questions or get help. Participants also considered risk scores being given by researchers over the phone as suitable. One male participant spoke in terms the current study engagement with mental health topics through contact with researchers: “I think we are prone to participate if it’s like we are doing right now.” (ID 3047, Male). At the same time, many participants voiced concerns about getting a risk score from a general practitioner as they perceived general practitioners as lacking time as well as knowledge specific to mental health. Further, general practitioners were perceived as too quick to rely on medication as a solution.

Participants conveyed the value of multiple methods of risk score delivery from which recipients of personalized depression scores could choose. A male participant illustrated this saying: “Whoever they are comfortable with, right? Like I wouldn’t be comfortable to talk to my doctor about it but I would be comfortable talking to a counselor about it.” (ID 20686, Male) Many participants believed that no matter how they received their risk score, there should always be a choice in whether or not they wish to receive their risk score.

#### Augmenting risk scores with resources, strategies and follow up

Many participants believed that along with the risk score, resources to help people understand their scores and take steps to improve their mental health would be valuable. Resources could be in a relatively straightforward form of receiving a paper or online copy of their risk information that could be kept and read in the future. One female participant remarked that when receiving a personalized risk score: “If they are holding it (personalized risk score) in their hand they might try it (strategies for improving mental health) right away.” (ID 20570, Female). Further, participants noted that receiving a graphic to help visualize their risk score could improve their understanding.

Participants also spoke of the value of personal contact follow up after receiving risk scores. Some indicated that having unscheduled “random” follow up calls would help them to further reflect on, and take steps to improve, their mental health. Participants believed that such follow up contact could afford a chance to ask questions and better understand what factors contribute to their risk score. Follow ups would also provide opportunities to discuss strategies for improving their mental health and obtain input on whether they are making the right changes: “Maybe some kind of follow up, like a little nudge, ‘hey have you been following up on any self help strategies or whatever.’ If yes then good for you, if not then why not.” (ID 20468, Male).

The value of providing resources for improving mental health were described. One male participant distinguished the value of resources that are systematic to follow: “I definitely think if there was a way to point people to some resources but like specifically like systematic resources. Stuff like, I don’t know, just like off of my top. I think CBT is like cognitive behaviour therapy is like systematic. Like you just do the steps 1,2 and 3 and you will get results.” (ID 20657, Male).

#### Targeting young people to increase awareness

Participants expressed that targeting younger adults and children would be beneficial to broad goals of preventing depression. Some participants reasoned that children and young adults were more susceptible to depression, would benefit from knowing their risk scores, and were more likely to take the risk scores and make changes to their lives. Comparatively, it would be harder for adults to change behaviors and habits. Male participant commented: “I believe that should be taught in high school in personal health course or you know, everything regarding peer pressure and drugs and alcohol and how those things all come into play as an adult … I think adults are less perceptive than young adults” (ID 20642, Male). Another participant also claimed “target adolescents and university youths because it is the period where people usually develop habits that are problematic later on.” (ID 20671, Male).

## Discussion

### Principal findings

This qualitative analysis provided important insight about high-risk individuals’ perceptions regarding receiving personalized depression risk information. Risk prediction algorithms for mental health are a new topic in health care research, this study is one of the few to investigate the perceptions about how using these algorithms will affect the population. The main motivation for receiving the risk score was out of curiosity and to improve self-awareness of mental health. Receiving the risk information can provide a new perspective to discuss mental health problems openly because many of the participants in the study were open to sharing their score with friends and family. Participants had mixed views about the utility of personalized depression risk information, and the utility largely depends on the context and situation in which individuals were embedded. The optimal delivery of the personalized risk score was with a counselor, either in person or on the telephone. Providing additional resources, more follow-up conversations, and a paper copy are strategies that may enhance depression risk communication.

### Comparing to current literature

As part of this RCT, we conducted a qualitative study on participant perspectives 1 month after receiving the personalized depression risk information at baseline [21]. Consistent with that qualitative study, participants had a positive view about the risk information and a majority of them (over 93%) were interested in knowing their risk. Importantly, the RCT found that providing personalized depression risk information does not have a negative impact on physical or mental health [21]. A qualitative study conducted by Bellon and colleagues [29] in the Spanish primary care setting reported the same results. The Spanish study also indicated that primary care patients preferred health care professionals providing resources and helping them understand ways to prevent depression. This is, in part, consistent with our results; participants are often interested in sharing their risk information with their family doctors, but only if their doctor is both aware that they have depression and has been providing treatment for depression. On the other hand, many participants in this study reported that they preferred to share the information with spouses, family members and friends whom they trust and can rely on; this demonstrates the acceptability of the personalized depression risk information by the participants and its potential broader health promotion impact.

### Implications

Understanding motivations behind receiving their risk score is an important step in understanding whether or not someone will seek help, what those help seeking behaviors will be and their effect on the individual [30]. Similarly to what was found in this study, people often seek out health information with the intent to make positive behavior changes to improve their health or prevent an illness [31].

The results of this qualitative study offered some important insights about the formats of delivery and how personalized depression risk information may be better communicated. Personalized depression risk scores are new to individuals in the community, and many have little knowledge about what constitutes “high risk”. As such, a large proportion of high-risk individuals either over or underestimate their risk of having depression [32]. To this end, providing a comparative risk (e.g., the average risk of having an MDE in the general population) may help address this issue and improve users’ understanding of their risk. Participants also strongly recommended providing resources and effective strategies for risk reduction. Pertinent research in cardiology and oncology has shown that effective risk communication often include the following components: (1) Individualized risk presented as an absolute risk, as opposed to a relative risk [33]. The risk information may also list the individual’s risk factors. (2) Appropriate format of presentation (e.g., graphic, visual). The format of presentation can influence the degree to which individuals perceive their risk and will affect behavioural change, and (3) providing clear evidence-based information on available choices. It is clear that experience in other medical disciplines resonates well with the views of the participants in this study.

Participants preferred the idea of receiving depression risk information from a counselor. Moreover, participants also endorsed follow-ups by the research team in order for individuals to have a chance to have questions answered, discuss potential risk factors, receive information about effective self-help strategies, as well as information about mental health resources. Risk communication in oncology and cardiology is delivered either through written materials or the Internet, or with professional guidance (e.g., in-person or telephone education and counselling by trained coaches or health professionals) [34, 35]. It is possible that depression risk communication guided by counselors or trained coaches may be more effective than un-guided risk communication in motivating behaviour change. Evidence of that was found in the Spanish study by Moreno-Peral and colleagues [36] where they investigated the effect of personalized risk of depression information guided by health care professionals on anxiety. They found that this guided information decreased the anxiety of the participants. The decision about using guided or un-guided format will have cost implication when it comes to large scale implementation. A cost-effectiveness study was completed in Spain using a guided format in the clinical setting; they found that there was not increase in cost for increased quality of life [37]. Future studies are needed to compare the cost-effectiveness of guided and un-guided risk communication interventions and their benefits.

This study has several limitations. First, this study was completed during the global COVID-19 pandemic, which may have affected the perceptions of the participant’s mental health. Second, participants were asked to recall information from their past which may be vulnerable to reporting and recall biases. As a means to decrease the chances for recall bias, we only included participants who has recently completed their 12-month assessment in the qualitative interviews. This study was conducted in Canada, among individuals who were at high risk of having depression. Therefore, the results of this qualitative study should be understood as specific to this context. Finally, the results were not validated by the participants because they were not able to read and comment on the results of the study. To address that limitation, the analysis was completed by two different people separately than compared.

With the emerging interests in applying risk predictive analytics and machine learning techniques in the realm of mental health, it is anticipated that more risk predictive models for mental health problems will be developed in the clinical arena or in the general population. Nevertheless, these risk predictive models can only become useful when the baseline risk is communicated to the target population and the information is being acted upon. This qualitative study shed light on user perceptions about personalized depression risk information and how it may be best communicated, offering important information for designing personalized risk communication tools not only for depression and other mental health problems.

## Data Availability

The data that support the findings of this study are available on request from the corresponding author [JW]. The data are not publicly available due to the information could compromise research participant privacy/consent.
